# A Parallel Multi-Modal Factorized Bilinear Pooling Fusion Method Based on the Semi-Tensor Product for Emotion Recognition

**DOI:** 10.3390/e24121836

**Published:** 2022-12-16

**Authors:** Fen Liu, Jianfeng Chen, Kemeng Li, Weijie Tan, Chang Cai, Muhammad Saad Ayub

**Affiliations:** 1 School of Marine Science and Technology, Northwestern Polytechnical University, Xi’an 710072, China; 2 College of Mathematics and Computer Science, Yan’an University, Yan’an 716000, China; 3 State Key Laboratory of Public Big Data, College of Computer Science and Technology, Guizhou University, Guiyang 550025, China

**Keywords:** multi-modal information fusion, semi-tensor product, emotion recognition, low-rank matrix

## Abstract

Multi-modal fusion can exploit complementary information from various modalities and improve the accuracy of prediction or classification tasks. In this paper, we propose a parallel, multi-modal, factorized, bilinear pooling method based on a semi-tensor product (STP) for information fusion in emotion recognition. Initially, we apply the STP to factorize a high-dimensional weight matrix into two low-rank factor matrices without dimension matching constraints. Next, we project the multi-modal features to the low-dimensional matrices and perform multiplication based on the STP to capture the rich interactions between the features. Finally, we utilize an STP-pooling method to reduce the dimensionality to get the final features. This method can achieve the information fusion between modalities of different scales and dimensions and avoids data redundancy due to dimension matching. Experimental verification of the proposed method on the emotion-recognition task using the IEMOCAP and CMU-MOSI datasets showed a significant reduction in storage space and recognition time. The results also validate that the proposed method improves the performance and reduces both the training time and the number of parameters.

## 1. Introduction

The multi-modal fusion technique refers to integrating the information of multiple modalities by classification or prediction [1]. It has turned out to be an increasingly interesting topic in artificial intelligence applications such as multimedia event detection [2,3], sentiment analysis [4,5,6], behavior recognition [7], cross-modal translation [8,9], multi-modal urban sound tagging [10], visual question answering (VQA) [1,11], and emotion recognition [12,13,14,15]. The multi-modal fusion technique performs better prediction than employing any unimodal information [3].

Emotion recognition is considered to be a hot research topic in the field of multi-modal fusion and aims to integrate video, audio, and text modalities by employing fusion strategies at feature, model, and decision levels [16]. Previous works [17,18] merged modalities in a straightforward way. They have demonstrated the performances of feature-level fusion in the emotion-recognition task that could not model the complicated relationships. Decision-level fusion in [19,20] is usually implemented by combining the individual classification scores and is therefore not able to well capture the mutual correlation among different modalities. In [21], model-level fusion was performed by hidden Markov models, which facilitated the establishment of optimal connections among modailites according to the maximum entropy principle and the maximum mutual information criterion.

Recently, unlike existing approaches, the tensor-product representations have been extensively used for the multi-modal emotion-recognition tasks due to their impressive capabilities to directly achieve dynamic interactions in both inter-modality and intra-modality [3,22,23,24]. Zadeh et al. [25] proposed the tensor fusion network (TFN), by using the tensor cross-product to calculate the interactions between different features and learning both inter-modality and intra-modality dynamics in an end-to-end manner. It performs fusion at the feature level. Unfortunately, as the characteristic dimension increases, the number of parameters in the model increases exponentially, leading to high computation and memory costs. To tackle this problem, the low-rank multi-modal fusion (LMF) [26], the multi-modal, factorized, bilinear pooling (MFB) model [27], the memory fusion network (MFN) [28], and the multi-modal transformer (MuLT) [14] have been proposed to further improve the processing efficiency and evaluation. Saurav Sahay et al. proposed the LMF-MulT [15] method, which builds up on the MuLT and applies transformers to fused multi-modal signals that aim to capture all inter-modal signals via the LMF. It is trained fast and uses few parameters. It performs fusion at the model or decision level. However, these methods must satisfy the limitation of the dimension-matching conditions in matrix multiplication. We notice that in real cases, the features of various modalities have different scales and dimensions. Consequently, this can result in the inability to calculate or the need to match dimensions when calculating, leading to data redundancy. Most importantly, huge amounts of data require expensive hardware to store; storage devices thus limit the applicability of such methods on resource constrained devices, such as mobile phones and wearable devices. There is an urgent need to reduce storage space and runtime to enable deployment on mobiles and under-resourced devices.

To solve the aforementioned problems, we introduce a generalization mechanism of the conventional matrix product [29] in MFM pooling for multi-modal fusion, i.e., the semi-tensor product (STP). The STP does not depend on the dimensionality of the operational matrices or tensors. Due to its flexibility, the STP has been used in many fields. In the compressed sensing technique [30], STP has been introduced to replace the conventional matrix product in the sampling model. For visual question answering [31], the block-wise operation of STP has been applied to multi-modal fusion. In digital watermarking, Chen et al. [32] proposed a general nonnegative matrix factorization based on STP.

In this paper, we propose a hierarchical fusion method named parallel, multi-modal, factorized bilinear pooling based on semi-tensor product (PFBP-STP). The proposed method improves the efficiency of fusion at the feature level and decision level for various text and audio/video-based tasks. More importantly, it can make information fusion between different scales and dimensions of modalities independent of the dimension matching conditions in matrix multiplication. We applied this computationally efficient and flexible method to the emotion-recognition task.

The main contributions of this paper can be summarized as follows:

(1) Multi-modal, factorized, bilinear pooling based on STP, which can avoid data redundancy due to dimension matching, and reduces the computational and memory costs.
(2) We proposed a parallel, multi-modal, factorized, bilinear pooling method based on STP which can capture the rich interactions between the features by hierarchical fusion, and which realizes the arbitrary combination and fusion of three modalities.
(3) Experimental evaluation of the proposed methodology on two multi-modal datasets.

## 2. Notation and Preliminaries

In this section, a new matrix product named the semi-tensor product (STP) [29] is briefly reviewed initially. It is a generalization of the traditional matrix product and is applicable for two matrices of arbitrary dimensions. In addition, this generalization ensures the availability of all fundamental properties of the conventional matrix product. Therefore, it has become a very powerful and convenient new mathematical tool for investigating many matrix-expression-related problems.

We provide some basic preliminaries of the STP [33,34], which serve as the necessary theoretical basis of the proposed method.

**Definition 1.**  *For two matrices $X\in R^{m\times n}$ and $Y\in R^{p\times q}$, the left STP is denoted by ⋉ and can be expressed as:*

(1) $$X⋉Y=\left(X⊗I_{t/n}\right)\left(Y⊗I_{t/p}\right)\in R^{\left(m\cdot t/n)\times (t/p\cdot q\right)}$$

*where ⊗ denotes the Kronecker product [35], considering t=lcm(n,p) is the least common multiple of n and p; $I_{t/n}$ and $I_{t/p}$ are identity matrixes.*

In Equation (1), set *n* = *p*. It is obvious that left STP reverts to ordinary matrix multiplication as X⋉Y=XY.

**Definition 2.**  *Given a non-negative matrix $Z_{+}^{k\times l}$, we aim to find two non-negative matrices $X\in R_{+}^{m\times n}$, $Y\in R_{+}^{p\times q}$, such that*

(2) $$Z_{+}^{k\times l}=X_{+}^{m\times n}⋉Y_{+}^{p\times q}$$

*where t=lcm(n,p) is the least common multiple of n and p; k=m·t/n and l=q·t/p.*

**Definition 3.**  *Let $X\in R^{NP}$ be a row and $W\in R^{P}$ be a column. We split X into equal-size blocks as $\left(X^{1},X^{2},\ldots ,X^{P}\right)$, such that $X^{i}\in R^{N}$, i=1,…,P defines the STP of X and W is denoted by X⋉W, which is given as*

(3) $$X⋉W=\sum_{i=1}^{P}X^{i}w_{i}\in R^{N}.$$

## 3. Methodology

In this section, we first describe the architecture of the proposed model. Then, we introduce the concept of STP to extend the idea of bilinear pooling. Finally, we propose a parallel, multi-modal, factorized, bilinear pooling method based on the STP (PFBT-STP).

### 3.1. Model Architecture

**Figure 1:**
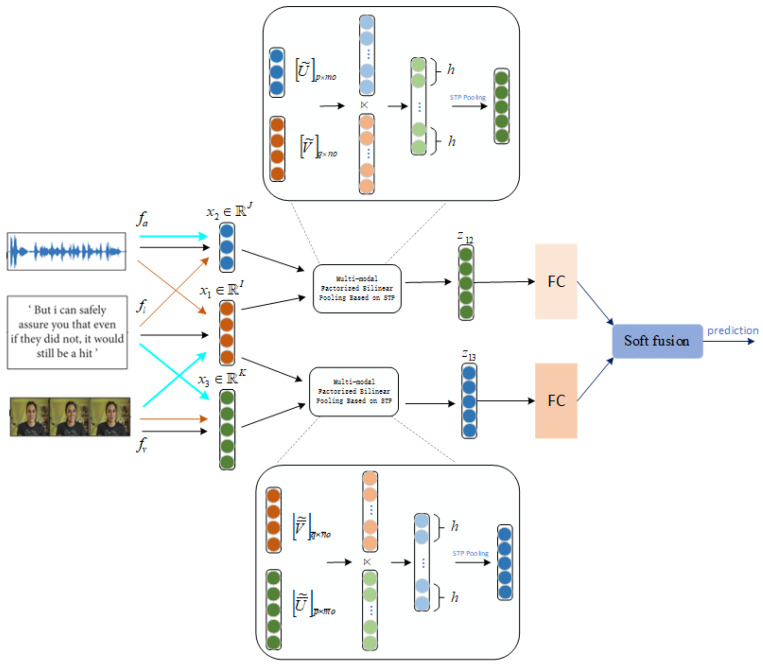
The basic architecture of our PFBP-STP method for emotion recognition.

Our method first obtains the unimodal representations $x_{1}\in R^{I}$, $x_{2}\in R^{J}$ and $x_{3}\in R^{K}$ by passing the unimodal inputs information (which includes text, video, and audio data) through three sub-embedding networks, $f_{l}$, $f_{a}$, and $f_{v}$, respectively. Then, we fuse one of these modalities with the other two modalities separately to exploit multi-modal, factorized, bilinear pooling based on STP, as the dimensionality differs among the features. Finally, we employ a decision fusion layer to improve the classification accuracy of the output features $z_{12}$ and $z_{13}$ for emotion recognition. The basic architecture of the PFBP-STP is shown in Figure 1.

### 3.2. Multi-Modal, Factorized Bilinear Pooling

In this section, we revisit the multi-modal bilinear models and the MFB pooling model.

Given two feature vectors $x_{1}\in R^{I}$ and $x_{2}\in R^{J}$, the multi-modal bilinear pooling is defined as follows:

(4) $$y_{i}=x_{1}^{T}W_{i}x_{2}$$

where $W_{i}\in R^{I\times J}$ is a projection matrix and $y_{i}\in R$ is the output of bilinear pooling. To obtain a *K*-dimensional output $y=\left[y_{1},\ldots ,y_{K}\right]$, a tensor $W=\left[W_{1},\ldots ,W_{K}\right]\in R^{I\times J\times K}$ needs to be learned. Unfortunately, the tensor W is a high-dimensional representation and introduces a larger number of parameters, which leads to a higher computation and memory cost and an even greater risk of overfitting, although multi-modal bilinear pooling can effectively capture the rich interactions between multi-modal features.

It is known from [36,37] that the low-rank approximation of non-negative matrix factorization can reduce the dimensionality of the original matrix, along with computational and memory costs. Hence, the two low-rank factor matrices have good interpretability, which is obtained by factorization.

Inspired by the matrix factorization techniques [37], the projection matrix $W_{i}$ in Equation (4) can be factorized into two low-rank matrices.

(5) $$W_{i}=U_{i}V_{i}^{T};$$

therefore, Equation (4) can be re-written as

(6) $$y_{i}=x_{1}^{T}U_{i}V_{i}^{T}x_{2}=\sum_{d=1}^{k}x_{1}^{T}u_{d}^{i}v_{d}^{i^{T}}x_{2}=1^{T}\left(U_{i}^{T}x_{1}\circ V_{i}^{T}x_{2}\right)$$

where *d* is the latent dimensionality of the factorized matrices $U_{i}=\left\lfloor u_{1}^{i},\ldots ,u_{d}^{i},\ldots ,u_{k}^{i}\right\rfloor \in R^{I\times k}$ and $V_{i}=\left\lfloor v_{1}^{i},\ldots ,v_{d}^{i},\ldots ,v_{k}^{i}\right\rfloor \in R^{J\times k}$, $1\in R^{k}$ is an all-one vector, and the operation ∘ represents the element-wise multiplication of two feature vectors or the Hadamard product.

According to Equation (6), we can get the following expression:

(7) $$y=\sum_{i=1}^{o}x_{1}^{T}U_{i}V_{i}^{T}x_{2}=x_{1}^{T}{\mathrm{UV}}^{T}x_{2}$$

where $y\in R^{o}$, and we need to learn two three-order tensors, i.e.,  $U=\left[U_{1},\ldots ,U_{o}\right]\in R^{I\times k\times o}$ and $V=\left[V_{1},\ldots ,V_{o}\right]\in R^{J\times k\times o}$, to obtain the output feature *y*. Generally, we reshape the tensors U and V as 2D matrices $\tilde{U}\in R^{I\times ko}$ and $\tilde{V}\in R^{J\times ko}$, respectively.

The fused (final) vector *z* can be obtained by summing non-overlapping windows of size *h* over the Hadamard product of projected matrices. We define the projections of feature vectors $x_{1}$ and $x_{2}$ in matrices $\tilde{U}$ and $\tilde{V}$ as ${\hat{x}}_{1}=\tilde{U}x_{1}$ and ${\hat{x}}_{2}={\tilde{V}}^{T}x_{2}$. We refer to the following model as the MFB pooling:

(8) $$z=\mathrm{SumPool}\left({\hat{x}}_{1}\circ {\hat{x}}_{2},k\right).$$

The above traditional multi-modal bilinear pooling method directly projects the features to the low-dimensional matrices and performs multiplication. In this process, there are two-dimensional matching conditions that must be satisfied in the matrix factorization and multiplication. In Equation (5), the projection matrix $W_{i}$ and two low-rank matrices $U_{i}$ and $V_{i}$ have to follow the dimension matching constraints.

In practice, the dimensions of multi-modal information, i.e., text, video, and audio, are different in the feature space. In this case, we need to match the dimensions, as it would cause data redundancy if we were to continue to use the traditional matrix factorization method.

### 3.3. Multi-Modal, Factorized Bilinear Pooling Based on STP

In order to solve the above problems, we propose a multi-modal, factorized bilinear pooling method based on STP. We factorize the projection matrix in Equation (5) by Definition 2 as follows:

(9) $$W_{i}=U_{i}⋉V_{i}^{T}$$

where $W_{i}\in R^{I\times J}$, $U_{i}\in R^{p\times m}$, and $V_{i}\in R^{q\times n}$. The variable t=lcm(n,p) is the least common multiple of *n* and *p*, I=p·t/m, and J=q·t/n. According to Equation (9), Equation (4) can be rewritten as

(10) $$y_{i}=x_{1}^{T}U_{i}⋉V_{i}^{T}x_{2}$$

further

(11) $$\begin{array}{cc} \; & y=\sum_{i=1}^{o}x_{1}^{T}U_{i}⋉V_{i}^{T}x_{2}=x_{1}^{T}U⋉V^{T}x_{2} \end{array}$$

where $y\in R^{o}$. We reshape the tensor $U=\left[U_{1},\ldots ,U_{o}\right]\in R^{p\times m\times o}$ and $V=\left[V_{1},\ldots ,V_{o}\right]\in R^{q\times n\times o}$ as 2D matrices $\tilde{\tilde{U}}\in R^{p\times mo}$ and $\tilde{\tilde{V}}\in R^{q\times no}$, respectively.

We define the projection of feature vectors $x_{1}$ and $x_{2}$ in matrices $\tilde{\tilde{U}}$ and $\tilde{\tilde{V}}$ as ${\bar{x}}_{1}=\tilde{\tilde{U}}x_{1}$ and ${\bar{x}}_{2}={\tilde{\tilde{V}}}^{T}x_{2}$. Similarly, Equation (11) can be re-written as

(12) $$y={\bar{x}}_{1}⋉{\bar{x}}_{2}$$

In addition, we propose a pooling method based on the STP (STP-pooling). The main function of pooling is reducing the dimensionality, which is achieved by the multiple dimension relation of the STP.

Let $y\in R^{1\times nh}$ and $w_{0}\in R^{h}$. We can split *y* into *n* equal-sized blocks as $y_{1},y_{2},\ldots ,y_{n}\in R^{1\times n}$. Then, the semi-tensor product can be represented as follows:

(13) $$y⋉w_{0}=\sum_{h=1}^{h}y_{h}w\left(h\right)\in R^{1\times n}$$

We get the final (fused) vector *z* by estimating the STP with a non-overlapping window of size *h* over the vector *y*.

(14) $$z=\mathrm{STP}-\mathrm{pooling}\;\left(y⋉w_{0},h\right)$$

In this section, the proposed method breaks the limitation of dimension matching conditions in matrix multiplication and achieves information fusion easily between different modalities having different scales and dimensions.

### 3.4. Parallel, Multi-Modal, Factorized Bilinear Pooling Based on STP (PFBP-STP)

Based on the above method, we arbitrarily merge the modalities of text, video, and audio at different scales to achieve multi-model fusion that overcomes the dimensional limitation in our task. The three modalities are represented as $x_{1}\in R^{I}$, $x_{2}\in R^{J}$, and  $x_{3}\in R^{K}$, respectively, and the fused features are denoted as $z_{12}\in R^{0}$ and $z_{13}\in R^{0}$, which represent the fusion results of $x_{1}$ with $x_{2}$ and $x_{3}$ respectively. Equation (11) can be rewritten as:

(15) $$y_{12}=\sum_{i=1}^{o}x_{2}^{T}U_{i}⋉V_{i}^{T}x_{1}=x_{2}^{T}U⋉V^{T}x_{1}$$

(16) $$y_{13}=\sum_{i=1}^{o}x_{3}^{T}{\bar{U}}_{i}⋉{\bar{V}}_{i}^{T}x_{1}=x_{3}{}^{T}\bar{U}⋉{\bar{V}}^{T}x_{1}$$

We reshape the tensors $U\in R^{p\times m\times o}$, $V\in R^{q\times n\times o}$, $\bar{U}\in R^{\bar{p}\times \bar{m}\times o}$, and  $\bar{V}\in R^{\bar{q}\times \bar{n}\times o}$ as 2D matrices $\tilde{U}\in R^{p\times mo}$, $\tilde{V}\in R^{q\times no}$, $\tilde{\bar{U}}\in R^{\bar{p}\times \bar{m}o}$, and  $\tilde{\bar{V}}\in R^{\bar{q}\times \bar{n}o}$ respectively.

Let us define the projections of feature vectors $x_{2}$ and $x_{3}$ on matrices $\tilde{U}$ and $\tilde{\bar{U}}$ as ${\bar{x}}_{2}=\tilde{U}x_{2}$ and ${\bar{x}}_{3}={\tilde{\bar{U}}}^{T}x_{3}$; meanwhile, we perform the projection of the feature vector $x_{1}$ on matrices $\tilde{V}\in R^{q\times no}$ and $\tilde{\bar{V}}\in R^{\bar{q}\times \bar{n}o}$ as ${\bar{x}}_{1}={\tilde{V}}^{T}x_{1}$ and ${\tilde{\bar{x}}}_{1}=\tilde{\bar{V}}x_{1}$ respectively. Similarly, we get $y_{12}={\bar{x}}_{2}⋉{\bar{x}}_{1}$ and $y_{13}={\bar{x}}_{3}⋉{\tilde{\bar{x}}}_{1}$. According to Equations (13) and (14), we get the final (fused) vectors $z_{12}$ and $z_{13}$ as follows:

(17) $$z_{12}=\text{STP-pooling}\;\left(y_{12}⋉w_{0},h\right)$$

(18) $$z_{13}=\text{STP-pooling}\;\left(y_{13}⋉w_{0},h\right)$$

The final (fused) vectors of $z_{12}$ and $z_{13}$ in Equations (17) and (18) are then fused via soft fusion at the decision-level stage to further improve the results. The weighted combination of the two groups of fusion modalities’ scores is mathematically shown as follows:

(19) $$S_{z}\left(c\right)=W_{12}\cdot S_{12}\left(c\right)+W_{13}\cdot S_{13}\left(c\right)\cdot$$

where $W_{12}$ and $W_{13}$ are the weights of $z_{12}$ and $z_{13}$. We set the same weight $W_{12}=W_{13}$ at the initial time. $S_{12}\left(c\right)$ and $S_{13}\left(c\right)$ represent the score matrices of $z_{12}$ and $z_{13}$ for the prediction of class *c*, and $S_{z}\left(c\right)$ stands for the final classification results. Algorithm 1 shows the process of PFBT-STP.
**Algorithm 1** PFBP-STP**Input:** 
vectors $x_{1}$, $x_{2}$ and $x_{3}$;
**Output:** 
vector $z_{12}$, $z_{13}$;
  1:Factorize the projection matrix: $W_{i}=U_{i}⋉V_{i}^{T}$.  2:Projecting: $\sum_{i=1}^{o}x_{2}^{T}U_{i}⋉V_{i}^{T}x_{1}$ and $\sum_{i=1}^{o}x_{3}^{T}{\bar{U}}_{i}⋉{\bar{V}}_{i}^{T}x_{1}$.  3:STP-pooling: *z*_12_ = STP-pooling $\left(y_{12}⋉w_{0},h\right)$, z_13_ = STP-pooling $\left(y_{13}⋉w_{0},h\right)$.  4:Soft fusion: $S_{z}\left(c\right)=W_{12}\cdot S_{12}\left(c\right)+W_{13}\cdot S_{13}\left(c\right).$  5:Return $S_{z}\left(c\right)$.


In this paper, we use the parallel, multi-modal, factorized, bilinear pooling method based on STP to fuse $x_{1}$ with $x_{2}$ and $x_{3}$, separately. It not only realizes the fusion of different scales and dimensions of information, but also avoids the problem of exponential growth when three modalities are fused simultaneously, leading to the risk of overfitting. This method also incorporates the scores from separate fusion modalities and generates a new prediction label by applying the soft fusion method at the decision-level fusion stage to further improve the results for the emotion-recognition task.

## 4. Experimental

In this section, we present various experiments to evaluate the characteristics of PFBP-STP and to support the following research claims:

(1) Comparison with state-of-the-art: We conducted experiments on PFBP-STP and state-of-the-art methods for an emotion-recognition task on IEMOCAP and CMU-MOSI datasets;
(2) The advantage of the PFBP-STP: It allows the information fusion independent of the dimension-matching conditions in matrix multiplication by replacing matrix products with semi-tensor products;
(3) Complexity analysis: We evaluate the speed and learned parameters of the method by comparing them with those of other methods.

### 4.1. Datasets

The proposed method was analyzed on the IEMOCAP [38] and CMU-MOSI [24] multi-modal datasets for emotion recognition.

The IEMOCAP dataset is designed to classify emotions such as voice and gesture displays during human interactions. It is an active, multi-modal, and multi-speaker database. It contains approximately 12 h of 302 videos. Each segment consists of nine different emotions: happy, angry, sad, excited, surprised, fear, neutral, frustrated, and disappointed. Ten actors performed three selected plays with clear emotional content. In addition to the script, subjects were asked to improvise conversations in hypothetical situations designed to elicit specific emotions (happy, angry, sad, depressed, and neutral states). Detailed motion-capture information, interaction configurations that elicit real emotions, and the size of the database make this corpus a valuable addition to existing databases to study and model multi-modal and expressive human communication.

The CMU-MOSI dataset is an opinion-level annotated corpus containing sentiment and subjectivity analysis of online videos such as YouTube videos. It includes 93 videos with comments. In each video, there are multiple opinion clips and emotional annotations within the range of [−3,3]. The two thresholds represent highly negative and highly positive opinions, respectively. For each video, an annotator was given 8 choices: highly negative (labeled as −3), negative (−2), weakly negative (−1), neutral (0), weakly positive (+1), positive (+2), and highly positive (+3). They could also choose to be “uncertain” in an ambigous situation. It not only has rigorous labels for sentiment intensity, subjectivity, visual features per-frame, and point of view, but also shows audio features per-millisecond.

The two datasets contain multiple information and were each divided into a training set, validation set, and test set to evaluate the generalization ability of the proposed model. It was ensured that there were no identical speakers between the training set and the test set. The data segmentation of the three sets is shown in Table 1.

### 4.2. Multi-Modal Data Features

The IEMOCAP dataset consists of three modalities, i.e., text, audio, and video. The unimodal features are extracted by utilizing global vectors for word representation, Glove [39], Facet, and COVAREP [40], respectively.

Text feature extraction implies Glove, an unsupervised learning algorithm that converts each word into a vector representation. For different inputs in the dataset above, the dimensions of each embedded text extracted by Glove number 300.

Audio feature extraction involved COVAREP, a collaborative and free speech-processing algorithm library. Low-frequency acoustic characteristics can be obtained by using COVAREP, including cepstrum coefficients of 12 MEL frequencies, tone tracking, glottic source parameters, glottic peak slope parameters, etc. Each audio feature was extracted with a 5 ms shift on a 25 ms frame, and each dataset has 74 dimensions.

Video features consist of 35 facial action units extracted using by Facet from each frame of the image. The video features are widely used to extract facial features, such as basic and advanced emotions. Thus, for each dataset, the dimensions of each video feature numbers 35.

### 4.3. Baseline

We choose early fusion LSTM (EF-LSTM) [14], late fusion LSTM (LF-LSTM) [14], the multi-modal transformer (MulT), [14], and the low-rank fusion-based transformer for multi-modal sequences (LMF-MulT) [15] as baselines. The MulT utilizes the low-rank representation of multi-modal sequences in the multi-modal transformer to pay cross-modal attention to modalities or fused signals. The LMF-MulT is built upon the MulT and applies transformers to fused multi-modal signals, aiming to capture all inter-modal signals through the low-rank matrix factorization (LMF).

### 4.4. Evaluation Metrics

In our experiments, multiple assessment tasks were performed, including regression and classification. The regression task was applied to CMU-MOSI. We used the accuracy Acc-*k* (where *k* represents the number of classes) and F1-score as the evaluation metrics for the CMU-MOSI. Specifically, for the other group, we used the 7-class accuracy (ACC-7) as the evaluation metric, which has seven sentiment scores. The mean absolute error (MAE) and the correlation (Corr) between the predicted results and the ground truth labels were used to evaluate the performance. The F1-score can be expressed by a weighted average of recall and precision as F1-score $=2\frac{\;\mathrm{precision}\;\cdot \;\mathrm{recall}\;}{\;\mathrm{precision}\;+\;\mathrm{recall}\;}$.

### 4.5. Training Setup

Our method was implemented on the open-source PyTorch framework. The hyper-parameters were selected using grid search, which is dependent on the performance of the model on the validation set. The sizes of video features, text features, and audio features were set to 35, 300, and 74, respectively. The parameters of the model used in training were configured as explained in the literature, where dropout was 0.2, weight normalization was $L_{2}$, and norm coefficient was 0.01. The Adam optimizer was employed with a learning rate of 0.0003, and the batch size was 32.

## 5. Results and Discussion

We present and discuss the experimental results in this section.

### 5.1. Comparison with the State-of-the-Art

We compared the performance of our model with those of the above baselines. The experimental results on the IEMOCAP and CMU-MOSI are presented in Table 2 and Table 3, respectively.

According to Table 2, the Acc values of the proposed method for happy, sad, and angry emotions were 85.7, 79.5, and 75.9, which are higher than the four baselines’ values. These observations indicate the necessity and effectiveness of applying STP in multi-modal fusion. Due to the multiple-dimension relation, STP is a block vs. block operation, unlike other mechanisms using point-wise operation. It keeps temporal and spatial information of the video, audio, and text, which allows for better representation of intra-modality correlations and improves the fusion performance.

From Table 3, it is shown that the Corr of the proposed method was 0.683, and the accuracy was 34.5% in the 7-class test, so it outperformed the baselines. In comparison with the performances of the baselines, the proposed method showed significant improvement on the IEMOCAP as compared to the CMU-MOSI.

### 5.2. Ablation Experiment

We chose bimodal or trimodal audio, video, and text as the input for emotion prediction. The bimodal and trimodal results are presented in Table 4.

For bimodal data, the experimental results in Table 4 demonstrate that the bimodal of t+v succeeded over the other two bimodals (a+t, a+v). This is because the audio contains some inevitable noises, and thus increases the difficulty of emotion recognition from speech. Compared with the audio, the text tends to have less noisy signals. Hence, we can learn more emotion-salient representation using text features.

Meanwhile, compared with bimodal inputs, the proposed method achieves better performance on trimodal inputs. Due to the complexity of emotion recognition, we can achieve better recognition performance by integrating multi-modal information.

### 5.3. Evaluation Indicators

Each modality of the central axis was combined and fused with the other two modalities, and the fusion results are compared under the three-layer framework. In this experiment, three modalities were randomly combined and fused, which demonstrates that the semi-tensor product is independent of the dimensional and scale-matching conditions for the fusion of the information using matrix multiplication.

We used the IEMOCAP dataset and the accuracy metrics to verify the performance of our method.

**Figure 2:**
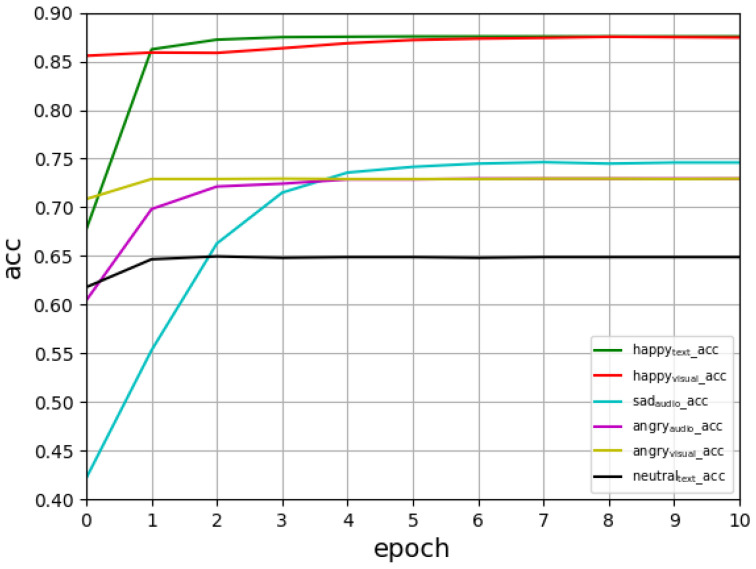
Comparison of the Acc of the three modalities with those of the other two modalities.

From Figure 2, we can see that the Acc score measure grows with each new iteration in the first seven epochs, which indicates that the model converges quickly.

**Figure 3:**
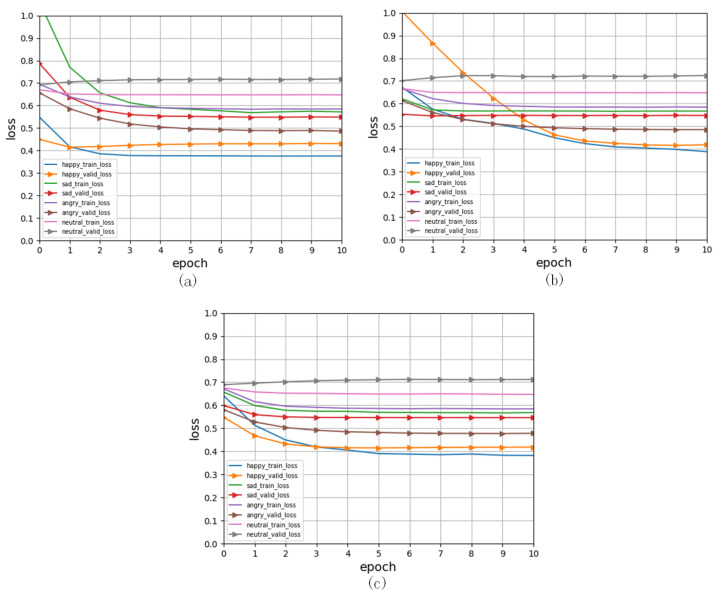
Training and validating measures are visible in the loss calculated for each iteration. (**a**). The fusion between audio with video and text. (**b**). The fusions between video and audio and text. (**c**). The fusions between text and audio and video.

In Figure 3, we can see that our model learns efficiently and quickly in training, and the loss function changes swiftly in the first five iterations and then smooths in the next iterations.

According to the accuracy values in Table 2, happy and angry emotions, again, had better recognition scores, whereas neutral had a worse recognition score during the training process; see Figure 2 and Figure 3. The main reason is that the model had a less obvious learning effect on attribute values with neutral features in IEMOCAP.

### 5.4. Computational Complexity

In order to evaluate the computational complexity of our method, we compared the parameters and the training speed of our method with those of MulT and LMF-MulT. The results are shown in Table 5 and Table 6.

In Table 5, we can observe that our model contained about 5.5 × 10^6^ parameters and MulT contains about 10.7 × 10^6^ parameters, which is almost twice the number. Experimental results show that the proposed method used less running time and fewer trainable parameters compared with the other two models to achieve better performance.

These models were all implemented in the same environment. Based on the results in Table 6, the proposed model significantly reduces the time required to train the model. Our model trained with an average time/epoch of 17.92 s on IEMOCAP and 17.92 s on CMU-MOSI. MulT trained at an average of 37.93 s per epoch on IEMOCAP and 19.25 s per epoch on CMU-MOSI, which is nearly 2 times slower.

Detailed analysis showed that the parameters are fewer and the running time is reduced, yet the performance of the original part is improved. This is due to the introduction of matrix decomposition based on the STP, which eliminates the data redundancy caused by the need for dimension matching for matrix factorization, and the three modalities of different dimensions can be arbitrarily combined and fused.

## 6. Conclusions

In this paper, a parallel, multi-modal, factorized, bilinear pooling method based on the semi-tensor product (PFBP-STP) is proposed, which achieves information fusion between modalities of different scales and dimensions. By replacing matrix products with STP, the information fusion becomes independent of the dimension-matching conditions in matrix multiplication.

Experiments have shown that the proposed method can achieve a significant increase in training speed and better classification accuracy simultaneously. The proposed method removes the dimensional consistency limitation of matrix multiplication and expresses the same information in a more compact structure that employ less memory. It is computationally friendly and flexible.

## Figures and Tables

**Table 1:** The data segmentation for training, validation, and test sets.

Dataset	IEMOCAP	CMU-MOSI
Training	6373	1284
Validation	1775	229
Test	1807	686

**Table 2:** Results for multi-modal emotion recognition on IEMOCAP.

Emotion	Happy		Sad		Angry		Neutral	
**Metric**	**F1**	**Acc**	**F1**	**Acc**	**F1**	**Acc**	**F1**	**Acc**
**EF-LSTM**	75.7	76.2	70.5	70.2	67.1	72.7	57.4	58.1
**LF-LSTM**	71.8	72.5	70.4	72.9	67.9	68.6	56.2	59.6
**MulT**	79.0	85.6	70.3	79.4	65.4	75.8	44.0	59.2
**LMF-MulT**	79.0	85.6	70.3	79.4	65.4	75.8	44.0	59.3
**OURS**	79.0	**85.7**	70.3	**79.5**	65.4	**75.9**	43.8	59.2

**Table 3:** Results for multi-modal emotion recognition on CMU-MOSI.

Metric	MAE	Corr	Acc-2	F1	Acc-7
**EF-LSTM**	1.078	0.542	73.6	74.5	31.0
**LF-LSTM**	0.988	0.624	77.6	77.8	33.7
**MulT**	1.008	0.645	80.3	80.4	34.3
**LMF-MulT**	0.957	0.681	78.5	78.5	34.0
**OURS**	1.038	**0.683**	71.7	78.9	**34.5**

**Table 4:** Performance of the proposed method using different modality combinations on IEMOCAP. Here, a, v, and t denote audio, video, and text, respectively.

Methods	Happy		Sad		Angry		Neutral	
	F1	Acc	F1	Acc	F1	Acc	F1	Acc
PFBT-STP (a+v)	77.9	85.2	69.2	79.3	65.4	75.8	42.5	57.9
PFBT-STP (a+t)	77.8	85.6	69.4	79.2	64.3	75.7	41.2	58.2
PFBT-STP (t+v)	78.5	85.7	70.0	79.5	65.1	75.9	43.7	58.8
PFBT-STP (a+v+t)	**79.0**	85.7	**70.3**	79.5	65.4	75.9	**43.8**	**59.2**

**Table 5:** Comparison of the number of parameters of our method with those of the other two models.

Dataset	IEMOCAP	CMU-MOSI
MulT	1074998	1071211
LMF-MulT	856078	512121
OURS	559872	500342

**Table 6:** Comparison of the average time per epoch for our method and the other two models.

Dataset	IEMOCAP	CMU-MOSI
MulT	37.93	19.25
LMF-MulT	23.53	12.03
OURS	17.92	11.92

## Data Availability

Not applicable.

## Abbreviations

The following abbreviations are used in this manuscript:

TFN	Tensor Fusion Network
EF-LSTM	Early Fusion LSTM
LF-LSTM	Late Fusion LSTM
MFB	Multi-Modal, Factorized Bilinear pooling
MuLT	Multi-modal Transformer
LMF-MulT	Low-Rank Fusion-based Transformer for Multi-modal Sequences
MFN	Memory Fusion Network
LMF	Low-rank Multi-modal Fusion
ACC	Accuracy
MAE	Mean Absolute Error

## References

[1] Baltrušaitis T., Ahuja C., Morency L.P. (2018). Multimodal machine learning: A survey and taxonomy. IEEE Trans. Pattern Anal. Mach. Intell..

[2] Habibian A., Mensink T., Snoek C. (2016). VideoStory Embeddings Recognize Events when Examples are Scarce. IEEE Trans. Pattern Anal. Mach. Intell..

[3] Shuang W., Bondugula S., Luisier F., Zhuang X., Natarajan P. (2014). Zero-Shot Event Detection Using Multi-modal Fusion of Weakly Supervised Concepts. Proceedings of the IEEE Conference on Computer Vision and Pattern Recognition.

[4] Park S., Han S.S., Chatterjee M., Sagae K., Morency L.P. Computational Analysis of Persuasiveness in Social Multimedia: A Novel Dataset and Multimodal Prediction Approach. Proceedings of the 16th International Conference on Multimodal Interaction.

[5] Zadeh A., Chen M., Poria S., Cambria E., Morency L.P. (2017). Tensor Fusion Network for Multimodal Sentiment Analysis. arXiv.

[6] Liu F., Chen J.F., Tan W.J., Cai C. (2021). A Multi-Modal Fusion Method Based on Higher-Order Orthogonal Iteration Decomposition. Entropy.

[7] Wu D., Chen J., Deng W., Wei Y., Luo H., Wei Y. (2020). The recognition of teacher behavior based on multimodal information fusion. Math. Probl. Eng..

[8] Qi J., Peng Y. (2018). Cross-modal Bidirectional Translation via Reinforcement Learning. Proceedings of the 27th International Joint Conference on Artificial Intelligence.

[9] Lee S., Kim I. (2018). Multimodal feature learning for video captioning. Math. Probl. Eng..

[10] Bai J.S., Chen J.F., Wang M. (2022). Multimodal Urban Sound Tagging with Spatiotemporal Context. IEEE Trans. Cogn. Dev. Syst..

[11] Guo W., Wang J., Wanga S. (2019). Deep Multimodal Representation Learning: A Survey. IEEE Access.

[12] Xie Z., Guan L. (2013). Multimodal Information Fusion of Audio Emotion Recognition Based on Kernel Entropy Component Analysis. Int. J. Semant. Comput..

[13] Pang L., Ngo C.W. (2015). Mutlimodal learning with deep boltzmann machine for emotion prediction in user generated videos. Proceedings of the 5th ACM on International Conference on Multimedia Retrieval.

[14] Tsai Y.H.H., Bai S., Liang P.P., Kolter J.Z., Morency L.P., Salakhutdinov R. (2019). Multimodal transformer for unaligned multimodal language sequences. Proceedings of the 57th Annual Meeting of the Association for Computational Linguistics.

[15] Sahay S., Okur E., Kumar S.H., Nachman L. (2020). Low rank fusion based transformers for multimodal sequences. arXiv.

[16] Zhou H., Du J., Zhang Y., Wang Q., Liu Q.F., Lee C.H. (2021). Information fusion in attention networks using adaptive and multi-level factorized bilinear pooling for audio-visual emotion recognition. IEEE/ACM Trans. Audio Speech Lang. Process..

[17] Mansoorizadeh M., Moghaddam Charkari N. (2010). Multimodal information fusion application to human emotion recognition from face and speech. Multimed. Tools Appl..

[18] Wang Y., Guan L., Venetsanopoulos A.N. (2012). Kernel cross-modal factor analysis for information fusion with application to bimodal emotion recognition. IEEE Trans. Multimed..

[19] Li S., Zheng W., Zong Y., Lu C., Tang C., Jiang X., Xia W. (2019). Bi-modality fusion for emotion recognition in the wild. Proceedings of the 19th International Conference on Multimodal Interaction.

[20] Liu C., Tang T., Lv K., Wang M. (2018). Multi-feature based emotion recognition for video clips. Proceedings of the 20th ACM International Conference on Multimodal Interaction.

[21] Zeng Z., Tu J., Pianfetti B.M., Huang T.S. (2008). Audio–visual affective expression recognition through multistream fused HMM. IEEE Trans. Multimed..

[22] Mai S., Hu H., Xing S. (2020). Modality to Modality Translation: An Adversarial Representation Learning and Graph Fusion Network for Multimodal Fusion. Proceedings of the 32th AAAI Conference on Artificial Intelligence.

[23] Fukui A., Park D.H., Yang D., Rohrbach A., Darrell T., Rohrbach M. (2016). Multimodal Compact Bilinear Pooling for Visual Question Answering and Visual Grounding. arXiv.

[24] Zadeh A., Zellers R., Pincus E., Morency L.P. (2016). MOSI: Multimodal Corpus of Sentiment Intensity and Subjectivity Analysis in Online Opinion Videos. arXiv.

[25] Zadeh A., Liang P.P., Poria S., Vij P., Morency L.P. (2018). Multi-attention Recurrent Network for Human Communication Comprehension. Proceedings of the 32 AAAI Conference on Artificial Intelligence.

[26] Liu Z., Shen Y., Lakshminarasimhan V.B., Liang P.P., Zadeh A., Morency L.P. (2018). Efficient Low-rank Multimodal Fusion with Modality-Specific Factors. arXiv.

[27] Yu Z., Yu J., Fan J., Tao D. (2017). Multi-modal factorized bilinear pooling with co-attention learning for visual question answering. Proceedings of the IEEE International Conference on Computer Vision.

[28] Zadeh A., Liang P.P., Mazumder N., Poria S., Morency L.P. (2018). Memory Fusion Network for Multi-view Sequential Learning. Proceedings of the Thirty-Second AAAI Conference on Artificial Intelligence.

[29] Cheng D. (2001). Semi-tensor product of matrices and its application to Morgen’s problem. Sci. China Ser. Inf. Sci..

[30] Fu W., Li S. (2018). Semi-Tensor Compressed Sensing for Hyperspectral Image. Proceedings of the IEEE International Geoscience and Remote Sensing Symposium.

[31] Bai Z., Li Y., Zhou M., Li D., Wang D., Połap D., Woźniak M. (2020). Bilinear Semi-Tensor Product Attention (BSTPA) model for visual question answering. Proceedings of the 2020 International Joint Conference on Neural Networks.

[32] Chen Z., Li L., Peng H., Liu Y., Yang Y. (2018). A novel digital watermarking based on general non-negative matrix factorization. IEEE Trans. Multimed..

[33] Cheng D., Qi H., Zhao Y. (2012). An Introduction to Semi-Tensor Product of Matrices and Its Applications.

[34] Cheng D., Qi H. (2010). A linear representation of dynamics of Boolean networks. IEEE Trans. Autom. Control.

[35] Tucker L. (1966). Some mathematical notes on three-mode factor analysis. Psychometrika.

[36] Liu W.H., Zhen N.N., You Q.B. (2006). Non-negative matrix factorization and its application in pattern recognition. Chin. Sci. Bull..

[37] Hubert L., Meulman J., Heiser W. (2000). Two purposes for matrix factorization: A historical appraisal. SIAM Rev..

[38] Busso C., Bulut M., Lee C.C., Kazemzadeh A., Mower E., Kim S., Chang J.N., Lee S., Narayanan S.S. (2008). IEMOCAP: Interactive Emotional Dyadic Motion Capture Database. Lang. Resour. Eval..

[39] Pennington J., Socher R., Manning C. (2014). Glove: Global Vectors for Word Representation. Proceedings of the 2014 Conference on Empirical Methods in Natural Language Processing.

[40] DeGottex G., Kane J., Drugman T., Raitio T., Scherer S. (2014). COVAREP: A Collaborative Voice Analysis Repository for Speech Technologies. Proceedings of the IEEE International Conference on Acoustics Speech and Signal Processing.

